# RETRACTION: Biochemical profile of milk thistle (*Silybum Marianum* L.) with special reference to silymarin content

**DOI:** 10.1002/fsn3.4170

**Published:** 2024-05-02

## Abstract

**RETRACTION:** Maryam Aziz, Farhan Saeed, Nazir Ahmad, Aftab Ahmad, Muhammad Afzaal, Shahzad Hussain, Abdellatif A. Mohamed, Mohamed S. Alamri, and Faqir Muhammad Anjum, “Biochemical profile of milk thistle (*Silybum Marianum* L.) with special reference to silymarin content,” *Food Science & Nutrition 9* (2021): 244–250, https://doi.org/10.1002/fsn3.1990.

The above article, published online on 09 November 2020 in Wiley Online Library (wileyonlinelibrary.com), has been retracted by agreement between the journal's Editor‐in‐Chief, Y. Martin Lo, and Wiley Periodicals, LLC. The retraction has been agreed to following concerns about the peer review process. The editorial office found unambiguous evidence that the manuscript was accepted solely based on compromised and insufficient reviewer reports. This evidence was further investigated and verified by the publisher and the Editor‐in‐Chief. As a result, the conclusions of this article are not considered reliable. The authors were notified of the retraction and disagreed with this decision.

**RETRACTION:** Maryam Aziz, Farhan Saeed, Nazir Ahmad, Aftab Ahmad, Muhammad Afzaal, Shahzad Hussain, Abdellatif A. Mohamed, Mohamed S. Alamri, and Faqir Muhammad Anjum, “Biochemical profile of milk thistle (*Silybum Marianum* L.) with special reference to silymarin content,” *Food Science & Nutrition 9* (2021): 244–250, https://doi.org/10.1002/fsn3.1990.

The above article, published online on 09 November 2020 in Wiley Online Library (wileyonlinelibrary.com), has been retracted by agreement between the journal's Editor‐in‐Chief, Y. Martin Lo, and Wiley Periodicals, LLC. The retraction has been agreed to following concerns about the peer review process. The editorial office found unambiguous evidence that the manuscript was accepted solely based on compromised and insufficient reviewer reports. This evidence was further investigated and verified by the publisher and the Editor‐in‐Chief. As a result, the conclusions of this article are not considered reliable. The authors were notified of the retraction and disagreed with this decision.

